# Data on leukocyte PDZK1 deficiency affecting macrophage apoptosis but not monocyte recruitment, cell proliferation, macrophage abundance or ER stress in atherosclerotic *plaques of LDLR deficient mice*

**DOI:** 10.1016/j.dib.2018.05.128

**Published:** 2018-05-26

**Authors:** Pei Yu, Alexander S. Qian, Kevin M. Chathely, Bernardo L. Trigatti

**Affiliations:** a Department of Biochemistry and Biomedical Sciences, McMaster University, Hamilton, Ontario, Canada L8S 4L8; b Thrombosis and Atherosclerosis Research Institute, McMaster University and Hamilton Health Sciences, Hamilton, Ontario, Canada L8S 4L8; c Medical Sciences Graduate Program, McMaster University, Hamilton, Ontario, Canada L8S 4L8

## Abstract

PDZK1 (Post-synaptic density protein/Drosophila disc-large protein/Zonula occludens protein containing 1) is an adaptor protein that binds to the HDL receptor, Scavenger receptor class B type I. Leukocyte PDZK1 deficiency in high fat-diet fed LDL receptor knockout mice has been found to increase atherosclerotic necrotic core formation and apoptosis of cells within atherosclerotic plaques. To explore mechanisms that may be involved, we examined the effects of leukocyte PDZK1 deficiency in mice on a number of processes that may impact macrophage abundance within atherosclerotic plaques. We found that leukocyte PDZK1 deficiency in high fat diet fed LDL receptor knockout mice did not affect the abundance of circulating red blood cells, myeloid cells or B- or T-lymphocytes. Leukocyte selective PDZK1 deficiency did not affect the levels of the ER chaperone proteins, detected with an antibody against the KDEL peptide, in macrophages or macrophage abundance, cellular proliferation or monocyte recruitment in atherosclerotic plaques. Leukocyte PDZK1 deficiency in otherwise wild type mice did result in increased sensitivity of macrophages to tunicamycin-induced apoptosis in a peritonitis model. HDL protected wild type macrophages from apoptosis induced by a variety of agents, including the ER stressor tunicamycin, oxidized LDL and exposure to UV irradiation. However, this protection afforded by HDL was lost when macrophages were deficient in PDZK1. HDL did not affect the level of ER stress induction by tunicamycin. Finally, PDZK1 deficiency in macrophages did not affect lipopolysaccharide-mediated induction of markers of M1 polarization. These data, utilizing mouse and cellular models, help to demonstrate that leukocyte PDZK1 plays a role in atherosclerosis by affecting macrophage apoptosis within atherosclerotic plaques.

**Specifications Table**

**Table:** 

Subject area	*Biology/Biomedical sciences*
More specific subject area	*Mechanisms of atherosclerotic human disease*
Type of data	*Graphs, figures*
How data was acquired	*Microscope (Zeiss Axiovert 200 M)*
Data format	*Analyzed*
Experimental factors	Mice underwent bone marrow transplantation
	Mice were fed a high fat diet
	Peritonitis was induced with 1 ml, 10% thioglycollate
	ER stress/UPR was induced in mice and cells with tunicamycin
	Cultured cells were treated with HDL and different apoptosis inducing agents
Experimental features	*Histological sections of atherosclerotic plaques and primary macrophages from experimental mice were used.*
Data source location	*Hamilton, Ontario, Canada*
Data accessibility	*Data included in this article and is related to articles published*

**Value of the data**

• The data presented herein is key to understanding the consequences of inactivating PDZK1 gene expression in bone marrow derived cells on atherosclerosis development.
• This data gives insight into mechanisms by which PDZK1 influences atherosclerosis development.
• This data provides a more thorough understanding of how PDZK1 protects macrophages against apoptosis induced by different stressors.

## 1 Data

### 1.1 Effects of bone marrow selective inactivation of PDZK1 on atherosclerotic plaques in *ldlr* KO mice

**Fig. 1:**
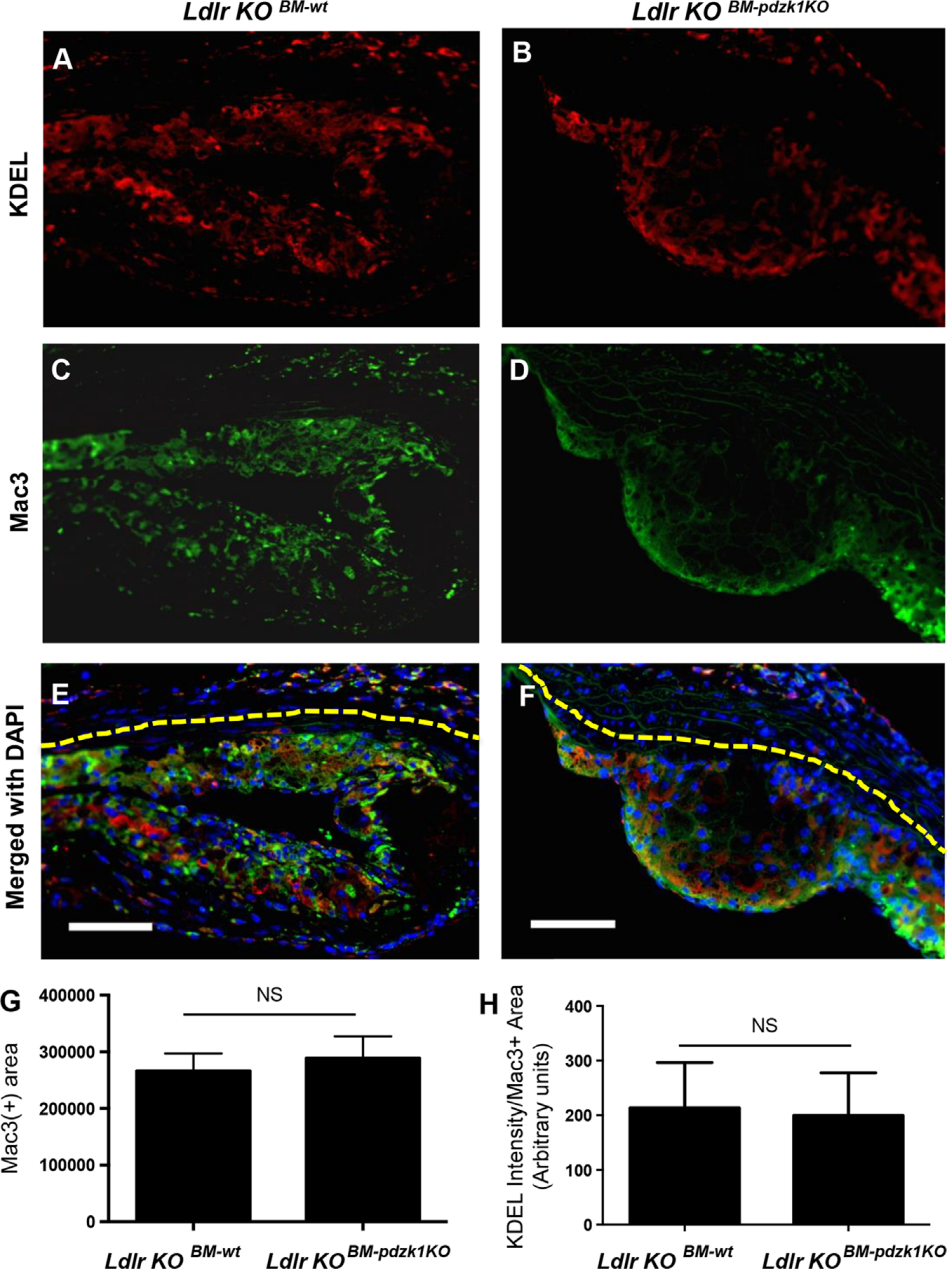
*Ldlr* KO mice transplanted with *wt* or *pdzk1* KO BM do not differ in macrophage abundance or ER stress in plaques. Cross-sections of aortic sinus atherosclerotic plaques from *ldlr* KO mice transplanted with *wt* (A, C, E, *n* = 6) or *pdzk1* KO (B, D, F, *n* = 4) BM were co-stained with anti-KDEL antibody (red), anti-Mac3 antibody (green) and DAPI (blue). (G) Quantification of the Mac-3^+^ area. (H) The intensity of anti-KDEL staining was determined using Image J software and normalized to the size of Mac-3^+^ area. Data was analyzed by the Mann-Whitney rank sum test. NS indicates not statistically significant (*p* = 0.6 for G and *p* > 0.99 for H).

**Fig. 2:**
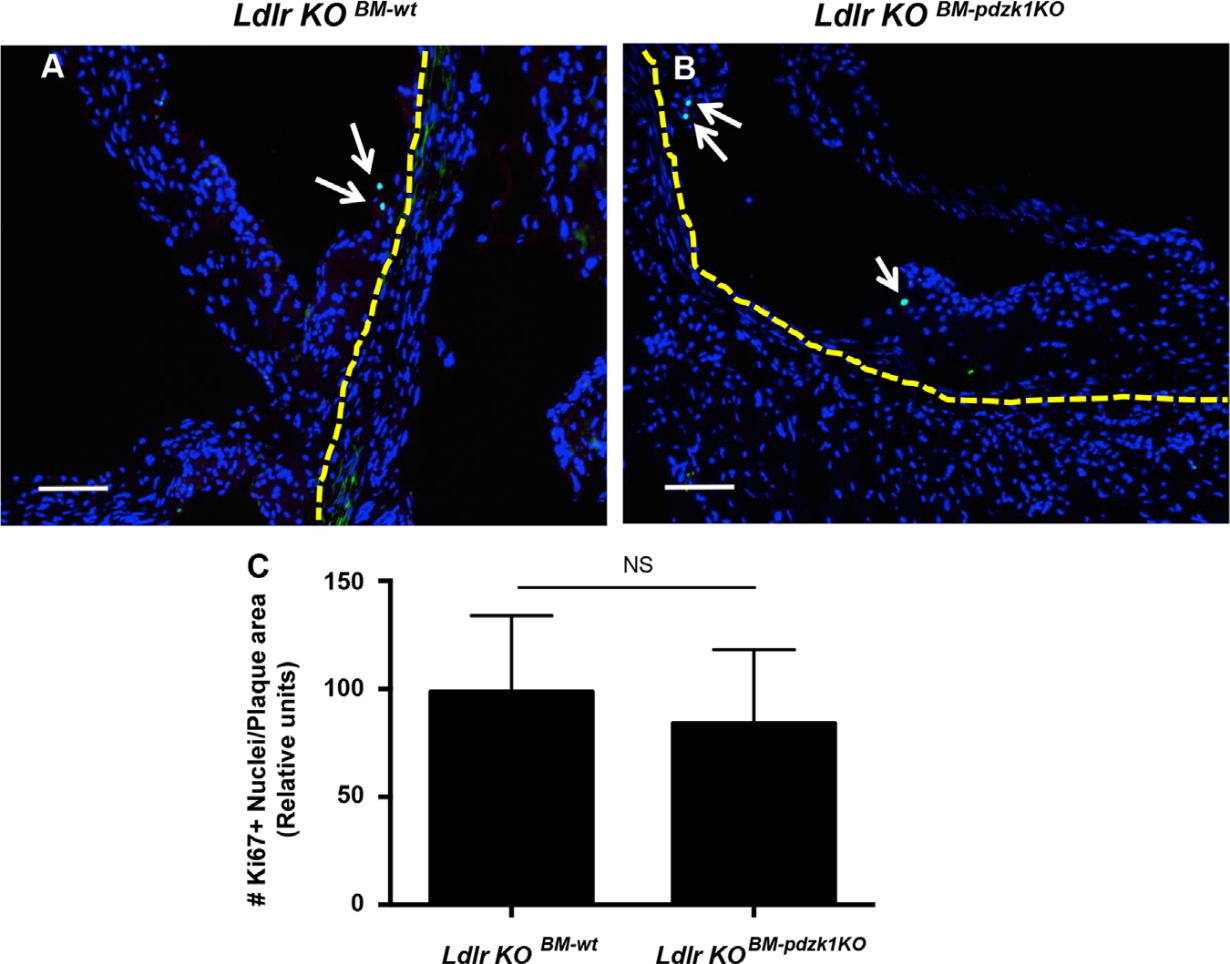
***Ldlr*****KO mice transplanted with*****wt*****or*****pdzk1*****KO BM showed similar levels of cell proliferation in atherosclerotic plaques.** Cross-sections of atherosclerotic plaques in the aortic sinus were stained for Ki67^+^ nuclei (green staining; indicated by white arrows) and counter-stained with DAPI (blue staining). The arterial wall is outlined by the yellow dashed line. Representative sections from mice transplanted with **(A)***wt* or **(B)***pdzk1* KO BM. Scale bar = 50 μm. **(C)** Quantification of Ki67^+^ nuclei in atherosclerotic plaques (*n* = 8 *wt* or 10 *pdzk1* KO BM transplanted mice), normalized to plaque area. Data was analyzed by the Mann-Whitney rank sum test. NS indicates not statistically significant (*p* = 0.47).

**Fig. 3:**
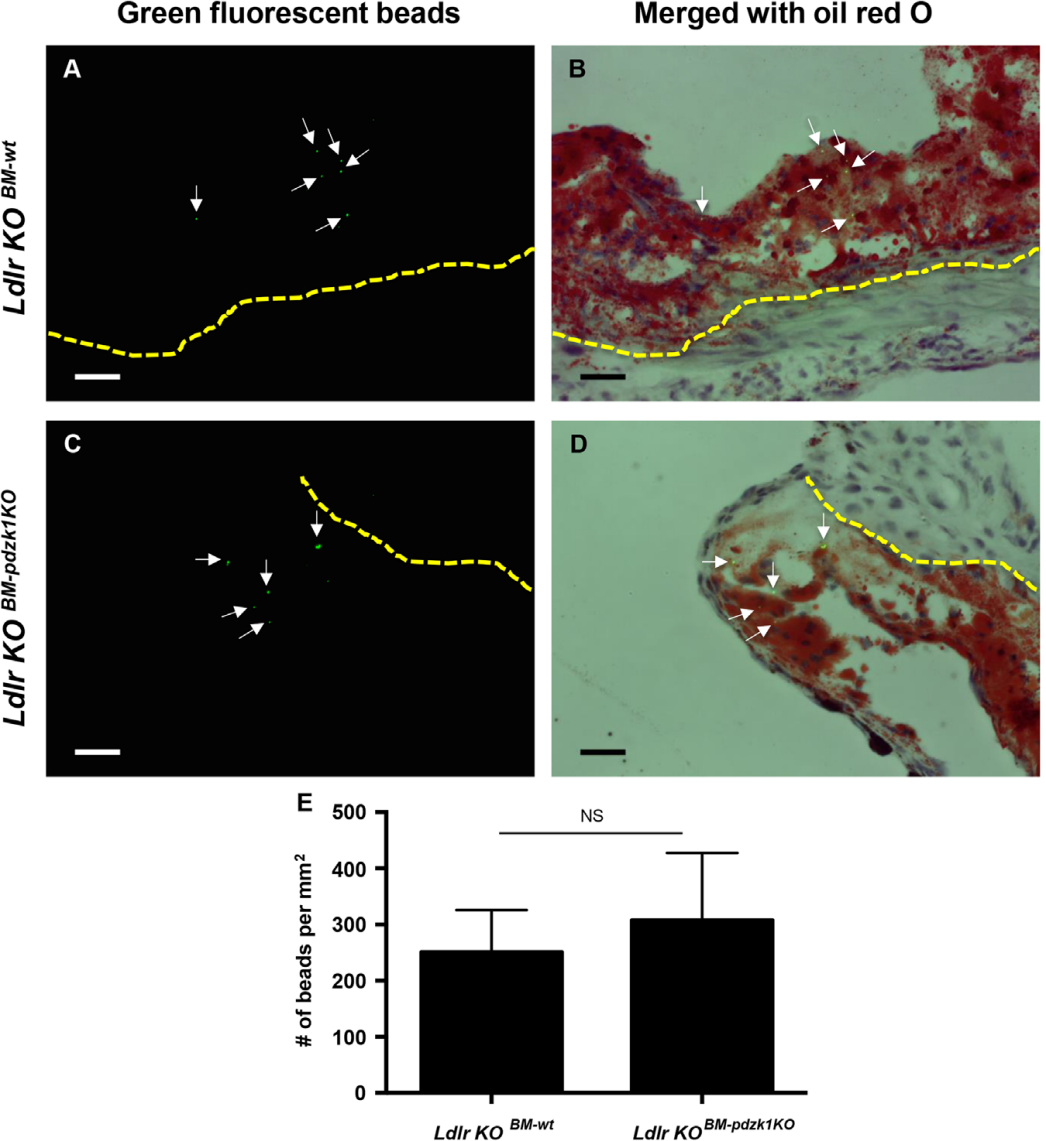
**Monocyte recruitment into atherosclerotic plaques is unaffected in*****ldlr*****KO mice by BM specific inactivation of*****pdzk1**. Ldlr* KO mice were transplanted with BM from either *wt* or *pdzk1* KO donors, and then fed the high fat atherogenic diet for 10 weeks as described in the methods section of Ref [1]. Mice were injected with FITC-conjugated plain microspheres to label monocytes, euthanized 24 h later and atherosclerotic plaques in the aortic sinus were analyzed. **(A-D)** Representative images of green fluorescent beads alone **(A, C)** or merged with bright field oil red O images **(B, D)** of atherosclerotic plaques from mice transplanted with *wt* (**A, B**) or *pdzk1* KO BM (**C, D**). Scale bar = 50 μm. **(E)** Quantification of phagocyte recruitment as the number of green fluorescent beads in atherosclerotic plaques, normalized to the total area of plaques for n = 9 *wt* or 10 *pdzk1* KO BM transplanted mice. Data was analyzed by the Mann-Whitney rank sum test. NS indicates not statistically significant (*p* = 0.96).

To determine the effects of bone marrow selective inactivation of PDZK1 on high fat diet induced atherosclerosis, low density lipoprotein receptor (*ldlr*) knockout (KO) mice were transplanted with bone marrow (BM) from either *pdzk1* KO or corresponding wild type (*wt*) mice, allowed to recover for 4 weeks and then fed a high fat diet for a further 10 weeks. BM-specific *pdzk1* deletion did not significantly affect hematocrits, red blood cell sizes, or proportions of leukocytes that were positive for CD3, B220 or CD11b (Table 1). We detected no differences in the extent of immunostaining with an antibody against the –KDEL endoplasmic reticulum (ER) retention peptide, which detects the major ER chaperones, as a measure of ER stress (Fig. 1A, B, H). Similarly, we detected no differences in macrophage abundance (Mac3 immunostaining (Fig. 1C, D, G)) or in cell proliferation (Ki67 staining; Fig. 2) or monocyte recruitment into plaques (Fig. 3). On the contrary, in a parallel study [1], we detected increased atherosclerotic plaque sizes and increased cell apoptosis within atherosclerotic plaques of *ldlr* KO mice transplanted with BM from *pdzk1* KO donors, and subsequently fed the high fat diet.

**Table 1:** Blood cell parameters.

	*wt* BM	*pdzk1*^*-/-*^ BM	p^a^
Hematocrit(%)^b^	46.0 ± 3.8 (*n* = 3)	36.8 ± 1.8 (*n* = 4)	0.057
Mean red blood cell volume (MCV/fL)^b^	50.1 ± 0.4 (*n* = 3)	44.2 ± 0.7 (*n* = 4)	0.057
Red blood cell distribution width (RDW/%)^b^	17.5 ± 0.2 (*n* = 3)	19.0 ± 0.5 (*n* = 4)	0.057
% CD3+ cells^c^	17.6 ± 1.8 (*n* = 9)	19.7 ± 0.9 (*n* = 11)	0.29
% B220+ cells^c^	47 ± 2.2 (*n* = 9)	53 ± 1.5 (*n* = 11)	0.064
% CD11b+ cells^c^	29.9 ± 2.5 (*n* = 9)	26.5 ± 1.6 (*n* = 11)	0.27

a Statistical analysis was done using the Mann-Whitney rank sum test.

b Hematocrit, MCV and RDW was analyzed by Hemavet analysis of whole blood

c % CD3^+^, B220^+^ and CD11b^+^ cells were determined by flow cytometry and are expressed as the proportions of total leukocytes.

### 1.2 BM selective inactivation of PDZK1 increases sensitivity of peritoneal macrophages to ER stress induced apoptosis

**Fig. 4:**
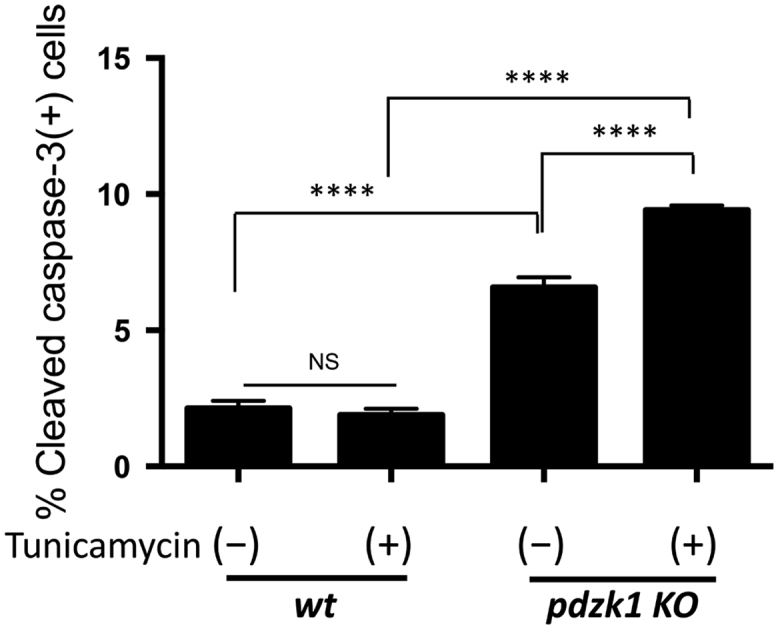
**in vivo tunicamycin-induced apoptosis of peritoneal macrophages in*****wt*****mice with BM-selective*****pdzk1*****deficiency.***Wt* mice were transplanted with BM from control *wt* (*n* = 6) or *pdzk1* KO donors (*n* = 9) and allowed to recover for 8 weeks. Mice were injected i.p. with 1 ml of 10% thioglycollate, to recruit macrophages. 72 h later, mice were injected i.p. with tunicamycin (1 mg/kg body weight) (*n* = 3 for mice with *wt* BM and *n* = 4 for mice with *pdzk1* KO BM) or an equivalent volume of DMSO as control (*n* = 3 for mice with *wt* BM and *n* = 5 for mice with *pdzk1* KO BM) and then euthanized 24 h later. Peritoneal macrophages were collected by peritoneal lavage, and subjected to CD11b, CC3 and PI staining and flow cytometry. Proportions of CD11b+ cells that were positive for CC3 staining were determined. No PI+ cells were detected. Data was analyzed by 1 way ANOVA with Tukey׳s multiple comparisons test. NS indicates not statistically significant (*p* = 0.95). ****p < 0.0001.

To test the effects of BM specific inactivation of PDZK1 on the sensitivity of macrophages to apoptosis, wild type mice were transplanted with BM from either *pdzk1* KO or control *wt* donors, allowed to recover for 8 weeks and then injected i.p. with thioglycollate to induce macrophage recruitment. Three days after thioglycollate injection, mice were injected i.p. with tunicamycin to induce ER stress and apoptosis in peritoneal cells. The next day, peritoneal cells were recovered, immunostained for apoptosis induction using an antibody for cleaved (activated) caspase 3 (CC3) and analyzed by flow cytometry (Fig. 4). We saw no induction of apoptosis by tunicamycin in peritoneal macrophages from mice transplanted with *wt* BM, but significant induction of apoptosis by tunicamycin in peritoneal macrophages from mice transplanted with *pdzk1* KO BM. Furthermore, we detected increased basal apoptosis in mice transplanted with *pdzk1* KO BM compared to mice transplanted with *wt* BM. In the accompanying article [1], we saw similar results for mice with whole body *pdzk1* KO compared to *wt* mice, although in that case, we detected no differences in basal apoptosis.

### 1.3 PDZK1 is required for HDL mediated protection against apoptosis induced by different agents

**Fig. 5:**
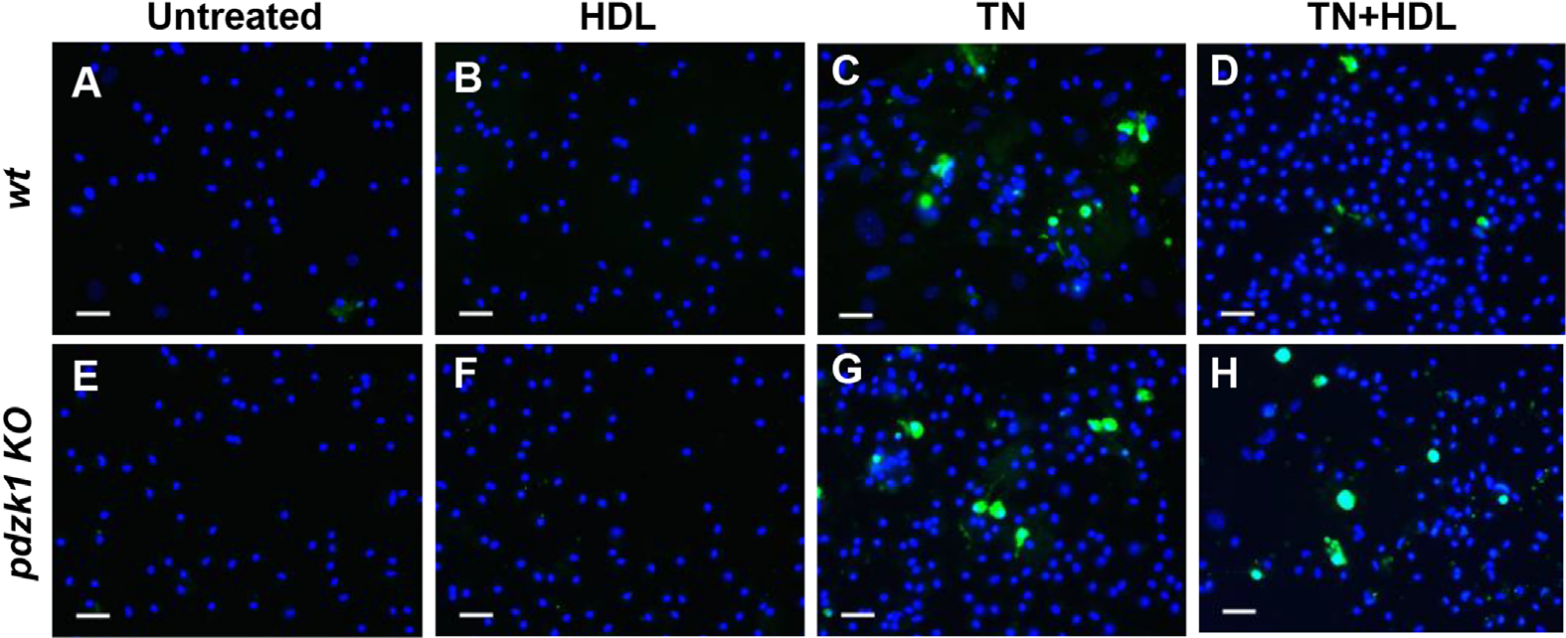
HDL protects *wt* but not *pdzk1* KO macrophages from tunicamycin-induced apoptosis measured by CC3 immunostaining. Peritoneal macrophages from *wt* or *pdzk1* KO mice were cultured in parallel in lipoprotein deficient medium and treated for 24 h with tunicamycin (TN) (10 μg/ml) or DMSO control in the presence or absence of HDL (50 μg protein/ml) as indicated. Apoptosis was detected by staining for CC3 (green). Nuclei were counterstained with DAPI (blue). Representative images of *n* = 3 are shown. Scale bars = 25 μm. Quantification is presented in Fig. 3I of Ref. [1].

**Fig. 6:**
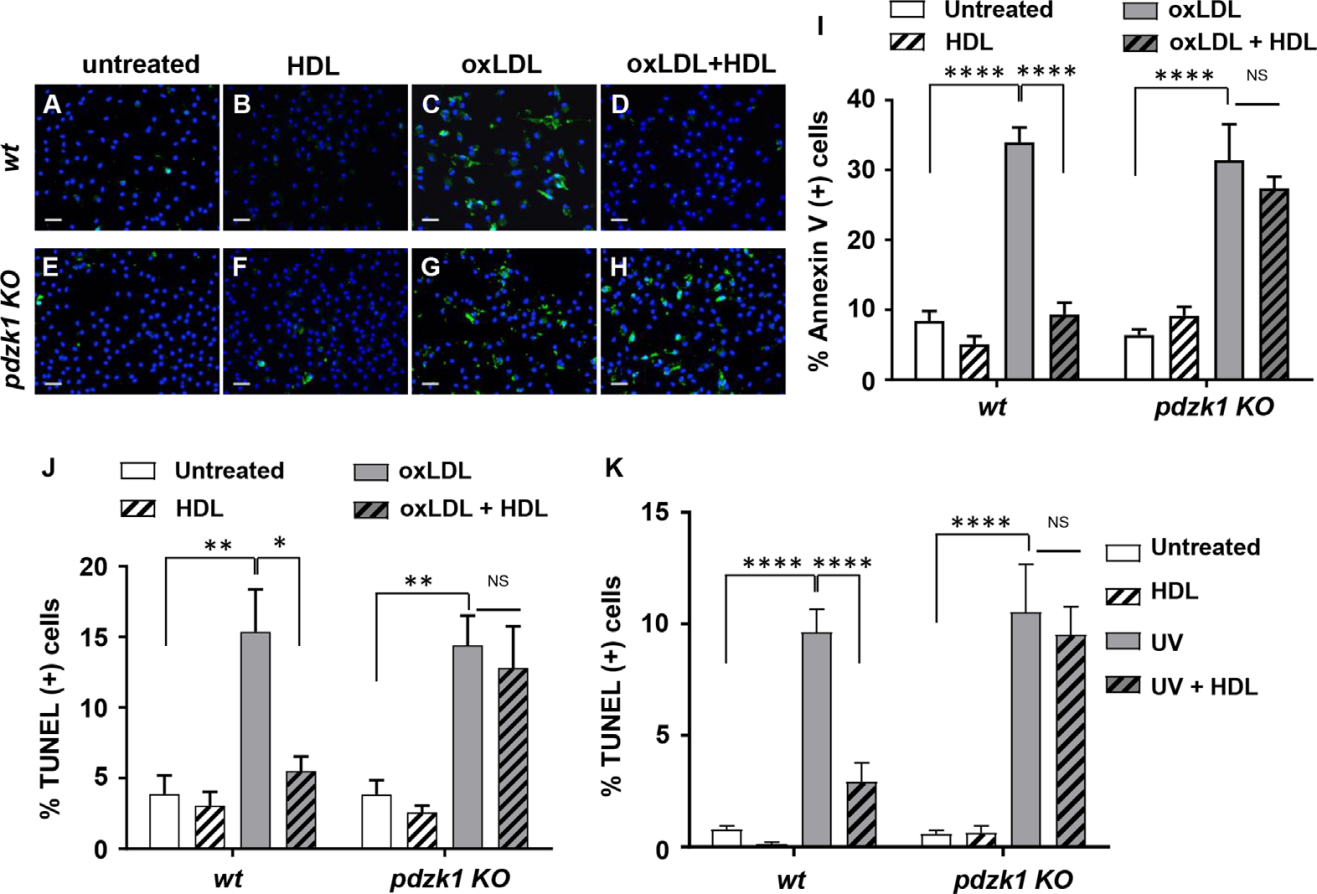
HDL protects *wt* but not *pdzk1* KO macrophages from apoptosis induced by oxLDL or UV irradiation. Peritoneal macrophages from *wt* or *pdzk1* KO mice were cultured in parallel in lipoprotein deficient medium and treated for 24 h with or without oxLDL (100 μg protein/ml) in the presence or absence of HDL (50 μg protein/ml) as indicated. Alternatively, cells were exposed to UV irradiation (50 mJ/cm^2^) and then treated for 24 h without or with HDL (50 μg protein/ml) as indicated. Apoptosis was detected by staining with FITC-annexin V or TUNEL. **(A-H)** Representative images of FITC-annexin V (green) and DAPI (blue) stained cells treated with oxLDL and HDL as indicated. Scale bars = 25 μm. **(I)** Quantification of % annexin-V positive cells after treatment with oxLDL and HDL as indicated. **(J)** Quantification of % TUNEL positive cells after treatment with oxLDL and HDL as indicated. (K) Quantification of % TUNEL positive cells after exposure to UV irradiation and treatment with HDL as indicated. Data are means ± SEM (*n* = 4 for data in I and J, *n* = 9 for data in K). Data was analyzed by 2 way ANOVA with Tukey׳s multiple comparisons test. NS indicates not statistically significant (*p* > 0.9). *p = 0.017; **p < 0.009; ****p < 0.0001.

Peritoneal macrophages were prepared from *wt* and *pdzk1* KO mice and analyzed in culture. Cells were treated with different apoptosis inducing agents, including tunicamycin, oxidized LDL (oxLDL) and exposure to UV irradiation. Apoptosis was measured by CC3 (Fig. 5), Annexin V (Fig. 6A-I) or TUNEL (Fig. 6J,K) staining. Fig. 5 shows representative images of *wt* and *pdzk1* KO macrophages that were either untreated or treated with tunicamycin in the absence or presence of HDL, prior to detection of apoptosis induction by staining for CC3. Data corresponding to these representative images was quantified and is presented as Fig. 3I in Ref [1]. We detected increased activation of caspase 3 in both *wt* and *pdzk1* KO macrophages treated with tunicamycin alone; furthermore, the extent of caspase 3 activation was suppressed in the presence of HDL in *wt* but not in *pdzk1* KO macrophages. Similarly, oxLDL increased apoptosis, measured as Annexin V cell staining, of both *wt* and *pdzk1* KO macrophages and HDL was able to suppress this in *wt* but not in *pdzk1* KO macrophages (Fig. 6 A-I). Similar results were obtained when apoptosis was induced by treatment with oxLDL (Fig. 6J) or exposure of cells to UV irradiation (Fig. 6K) and apoptosis was measured by TUNEL staining.

### 1.4 HDL treatment of macrophages does not prevent tunicamycin mediated induction of markers of ER stress/unfolded protein response

**Fig. 7:**
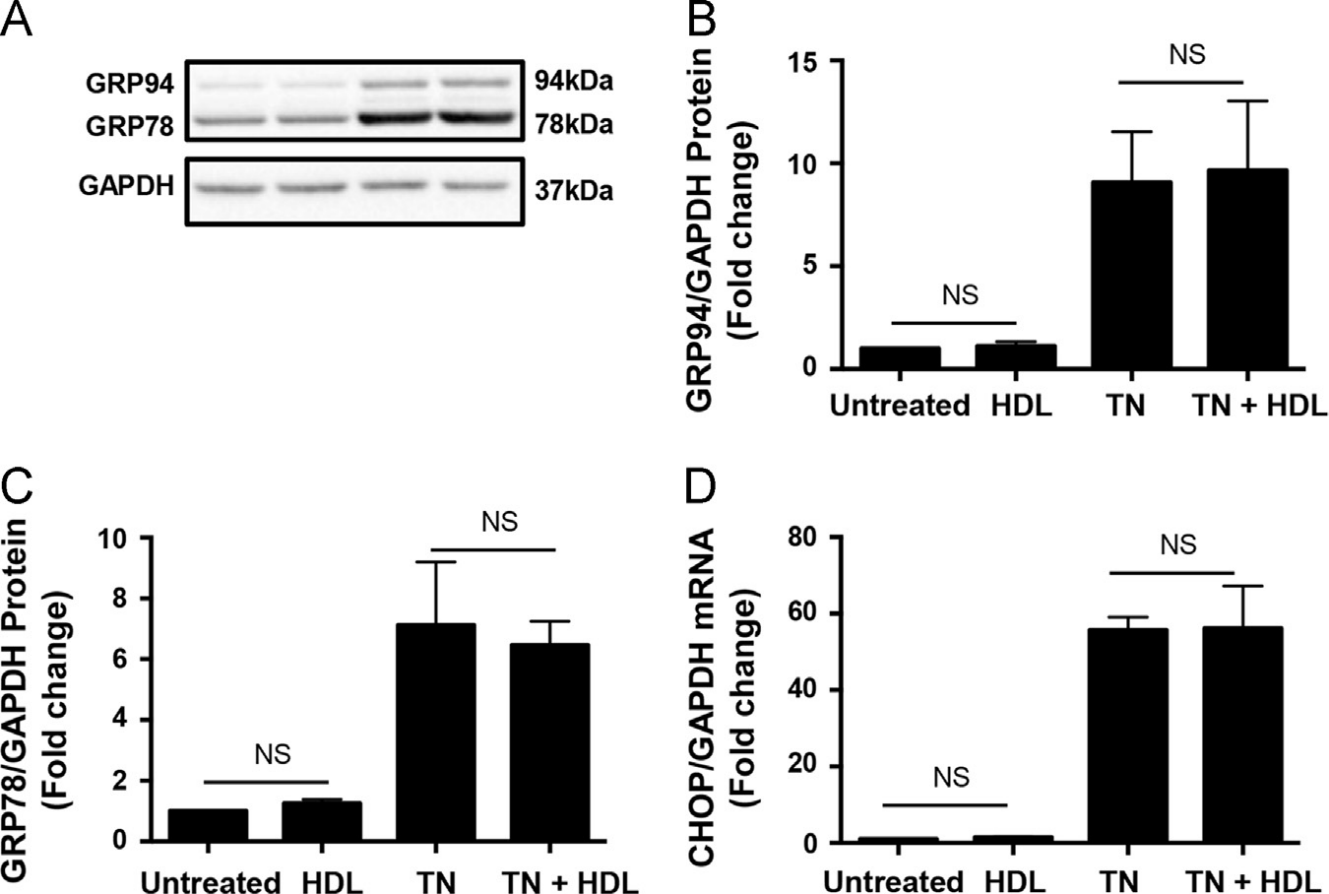
HDL does not prevent tunicamycin-mediated induction of markers of the ER unfolded protein response. Thioglycollate-elicited peritoneal macrophages were cultured in lipoprotein-deficient medium and treated with or without tunicamycin (10 μg/ml) for 24 h. in the presence or absence of HDL (50 μg protein/ml) as indicated. Cells were lysed and either subjected to SDS-PAGE for immunoblotting or RNA was extracted for RT-PCR analysis. **(A)** Representative immunoblots of GRP94 and GRP78, detected with an antibody against the –KDEL ER retention signal, and GAPDH (loading control). Quantification of **(B)** GRP94, and **(C)** GRP78, expressed as fold change relative to GAPDH. **(D)** Relative levels of CHOP mRNA normalized to GAPDH mRNA detected by RT-PCR. Data are means ± SEM (*n* = 3) and were analyzed by 1 way ANOVA with Tukey׳s multiple comparisons test. NS indicates not statistically significant (*p* > 0.97).

Treatment of macrophages with tunicamycin triggered caspase 3 activation (Fig. 5) and apoptosis [1] and this could be inhibited by HDL treatment of *wt* macrophages. Because tunicamycin is known to trigger apoptosis as a result of the induction of ER stress and the unfolded protein response [2], we tested if HDL treatment affected the ability of tunicamycin to induce markers of ER stress/unfolded protein response in *wt* macrophages (Fig. 7). We saw robust induction of the glucose regulated proteins of 94 and 78 kDa (GRP94 and GRP78) protein levels and of the mRNA for the C/EBP homologous protein (CHOP) in cells treated with tunicamycin. HDL treatment, however did not affect the ability of tunicamycin to induce these markers of ER stress/unfolded protein response (Fig. 7).

### 1.5 HDL does not induce STAT3 phosphorylation at the concentrations effective at protecting against apoptosis

**Fig. 8:**
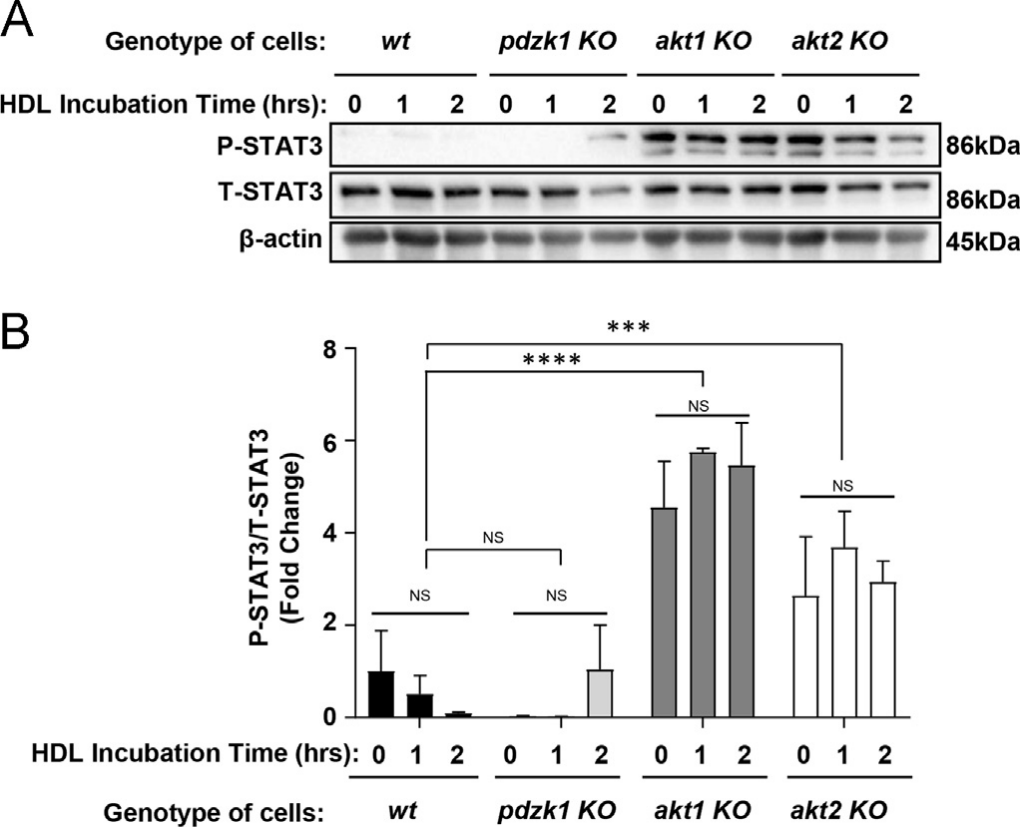
HDL treatment at 50 µg protein/ml does not induce STAT3 phosphorylation at Y705 in mouse peritoneal macrophages. Peritoneal macrophages from *wt*, *pdzk1* KO *akt1* KO and *akt2* KO mice were cultured in media containing lipoprotein deficient serum and treated with HDL at 50 µg protein /ml (to match conditions in which HDL protected against apoptosis in Fig. 5, Fig. 6), for 1 or 2 h. Control samples (0 time) were not treated with HDL. (A) Representative immunoblots of phospho-STAT3 (Y705) (P-STAT3), total-STAT3 (T-STAT3) and β-actin. (B) Quantification of the extent of STAT3 Y705 phosphorylation as P-STAT3/T-STAT3 band intensities (represented as fold change from *wt* cells at time 0) for *n* = 3 replicates. Data are means ± SEM. NS indicates no statistically significant differences between samples treated with HDL for different times (*p* > 0.9) as analyzed by 2 way ANOVA with Tukey׳s multiple comparisons test. Aggregate analysis of all of the samples from each genotype (regardless of HDL incubation time) revealed no statistically significant differences between *wt* and *pdzk1* KO macrophages (*p* = 0.99) but suggested increased P-STAT3 (Y705) in *akt1* KO and *akt2* KO compared to *wt* macrophages. **** indicates *p* < 0.0001; *** indicates *p* < 0.004 by 1 way ANOVA with Tukey׳s multiple comparison test of all samples from each genotype, removing HDL incubation time as a variable.

In the accompanying paper [1] we report that HDL treatment of peritoneal macrophages from *wt* but not *pdzk1* KO mice induced increased AKT phosphorylation. Because the signal transducer and activator of transcription 3 (STAT3) has been implicated by others [3] in HDL-mediated protection of RAW264.7 macrophages against apoptosis, we tested the effects of treatment of mouse peritoneal macrophages with HDL at 50 µg protein /ml, a concentration effective at protecting against apoptosis (Fig. 5, Fig. 6), on the levels of STAT3 phosphorylation. We saw that treatment with 50 µg/ml HDL did not significantly affect the levels of STAT3 phosphorylation at Y705 (reported to induce STAT3 dimerization [4]) in *wt* macrophages or in macrophages from *pdzk1* KO mice (Fig. 8). We also tested macrophages from *akt1* KO or *akt2* KO mice. Again, HDL treatment did not increase STAT3 Y705 phosphorylation. However baseline STAT3 Y705 phosphorylation appeared to be increased in *akt1* KO and *akt2* KO macrophages compared to wt macrophages (Fig. 8).

### 1.6 *Pdzk1* KO macrophages do not exhibit evidence of increased necroptosis induction

**Fig. 9:**
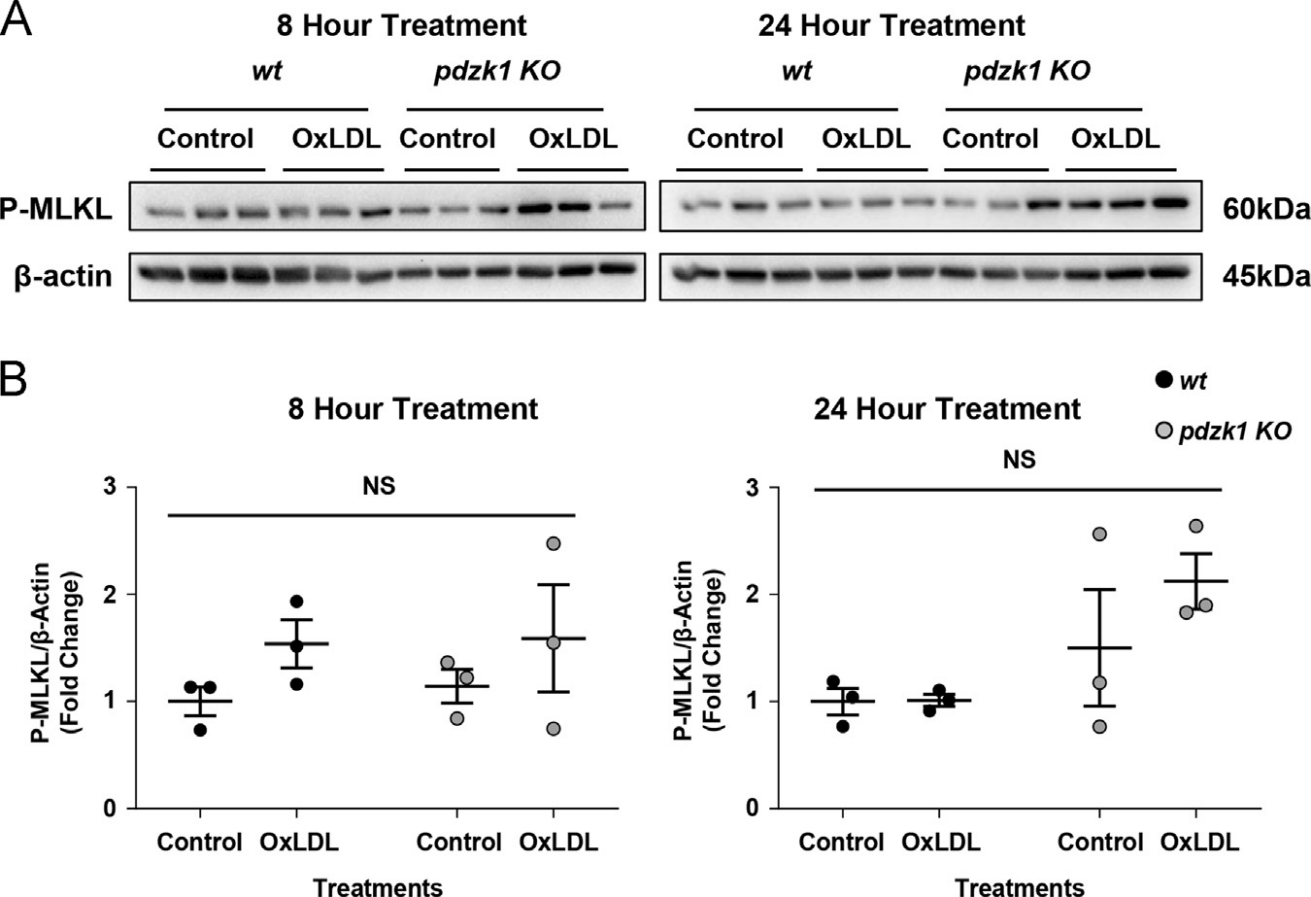
Phospho-MLKL levels after treatment of *wt* or *pdzk1* KO macrophages with oxLDL alone. Peritoneal macrophages from *wt or pdzk1* KO mice were cultured in media containing lipoprotein deficient serum and treated with oxLDL at 100 µg protein/ml or without oxLDL (control) for either 8 or 24 h. **(A)** Immunoblots of phospho (P)-MLKL and β-actin. **(B)** Quantification of the relative amount of P-MLKL expressed as the ratio of the band intensities for P-MLKL and β-actin (fold change relative to *wt* control cells). Each symbol represents a different replicate. Horizontal bars indicte the mean and error bars represent SEM. NS indicates no statistically significant differences (*p* > 0.5) as analyzed by 2 way ANOVA with Tukey׳s multiple comparisons test (*n* = 3 per group).

It has been reported that oxLDL treatment of macrophages can, under certain circumstances, trigger the induction of necroptosis, or programmed necrosis [5]. This involves the phosphorylation of the receptor interacting protein (RIP) kinases RIP1K and RIP3K and of mixed lineage kinase domain like (MLKL), which, upon phosphorylation, inserts into the plasma membrane and oligomerizes to form pores, causing cellular necrosis [6]. We therefore examined the level of phosphorylated MLKL in *wt* and *pdzk1* KO macrophages treated with oxLDL (100 µg protein/ml) for either 8 h or 24 h, corresponding to the conditions under which we observed that oxLDL triggered increased TUNEL, Annexin V (Fig. 6A–J) and cleaved caspase 3 staining [1]. After 8 h of oxLDL treatment average levels of phospho-MLKL tended to be higher in both *wt* or *pdzk1* KO macrophages, however the differences did not reach statistical significance. After 24 h of oxLDL treatment, phospho-MLKL levels were unchanged in *wt* macrophages and tended to be higher in *pdzk1* KO macrophages but, again, the results did not reach statistical significance (Fig. 9). This is consistent with reports that oxLDL induces necroptosis in the context of apoptosis inhibition (e.g. treatment with the pan-caspase inhibitor peptide zVAD-FMK) [5]. This suggests that in our studies and those reported in [1], treatment of macrophages with oxLDL in the absence of other agents (apoptosis inhibitors) led to induction of apoptosis but not necroptosis. We also saw an apparent trend towards increased levels of phospho-MLKL in *pdzk1* KO compared to *wt* macrophages in the 24 h treatment samples that was not apparent in the 8 hr treatment samples, however the results did not reach statistical significance (Fig. 9). Whether PDZK1 affects necroptosis induction under conditions which have been reported by others [5] to induce necroptosis (namely oxLDL treatment in the presence of apoptosis inhibition with zVAD-FMK) remains to be determined.

**Fig. 10:**
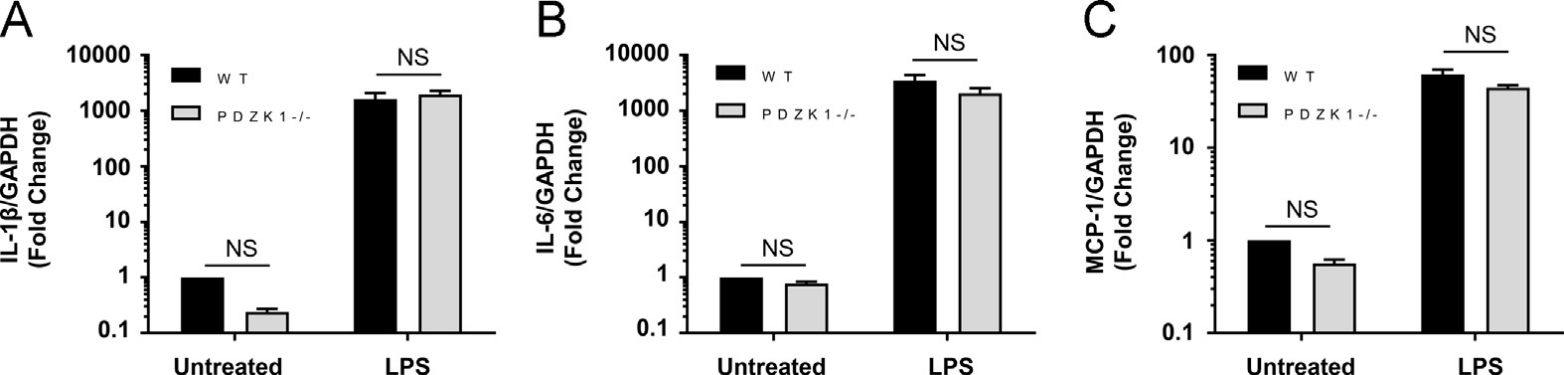
*Pdzk1* deficiency does not affect LPS-induction of markers of M1 polarization in macrophages. Quantitative RT-PCR for **(A)** IL-1β, **(B)** IL-6 and **(C)** MCP-1 in thioglycollate-elicited peritoneal macrophages from *wt* (black bars) or *pdzk1* KO mice (grey bars), treated in culture with 10 ng/ml LPS for 6 h. GAPDH was used as a control and data is presented as mean ± SEM fold change (n = 4), relative to untreated *wt* cells. Data was analyzed by 2 way ANOVA with Tukey multiple comparisons test. NS indicates no statistically significant differences: *p* > 0.27 for panels A and B and *p* > 0.06 for panel C.

### 1.7 Inactivation of PDZK1 in macrophages does not affect bacterial lipopolysaccharide (LPS) mediated induction of markers of M1 macrophage polarization

Peritoneal macrophages from *wt* or *pdzk1* KO mice were treated in culture without or with LPS and the expression of transcripts corresponding to inflammatory markers were determined by RT-PCR (Fig. 10). LPS treatment of both *wt* and *pdzk1* KO macrophages resulted in induction of interleukin (IL)-1β and IL-6 and monocyte chemotactic protein (MCP)-1 gene expression and inactivation of *pdzk1* did not alter this level of induction, suggesting that PDZK1 did not affect macrophage polarization towards an M1 phenotype.

## 2 Experimental design, materials and methods

### 2.1 Materials

OxLDL (#J5591/BT-910) and HDL (#J64903/BT-914) were from human sources and were purchased from Alfa Aesar (Tewksbury, MA, USA). Tunicamycin and LPS (from *E. coli* O111:B4) were from Sigma Aldrich Chemical Co (St. Louis, MO, USA). Antibodies and suppliers are listed in Table 2. All other reagents were obtained as described [1].

**Table 2:** Antibodies used in this study. IF indicates immunofluorescence, Flow indicates flow cytometry.

**Antibody**	**Supplier**	**Cat #**		**Use**
**Primary**				
HRP-anti-β-actin	Cell Signaling Technologies	5125 S	Immunoblot	
	Danvers, MA, USA			
Rabbit anti-cleaved caspase 3 (Asp175)	Cell Signaling Technologies	9661-S	IF, Flow	
	Danvers, MA, USA			
FITC rat anti-mouse CD3	BD Pharmingen	555274	Flow	
	Mississauga, ON, Canada			
APC rat anti-mouse CD11b (M1/70)	Life Technologies Inc.	RM2805	Flow	
	Burlington ON, Canada			
PerCP-Cy5.5 anti-hu/mo CD45R/B220	eBioscience, Thermo Fisher Scientific, Ottawa, ON, Canada	45–0452-80	Flow	
Rat anti-mouse CD107b (Mac3)	BD Biosciences, San Jose CA, USA	553322	IF	
HRP-anti-GAPDH	Abcam Inc	Ab9482	Immunoblot	
	Toronto, ON, Canada			
Mouse anti-mouse KDEL	Enzo Life Sciences Inc.,	SPA-827-D	Immunoblot, IF	
	Farmingdale NY, USA			
Rabbit anti-Ki67 (SP6) mAb	Abcam Inc.	ab16667	IF	
	Toronto ON, Canada			
Rabbit anti-phospho-MLKL (Thr 357/Ser 358)	Cell Signaling Technologies	14516	Immunoblot	
	Danvers, MA, USA			
Rabbit anti-phospho STAT3 (Tyr 705)	Cell Signaling Technologies	9131	Immunoblot	
	Danvers, MA, USA			
Rabbit anti-phospho STAT3 (Ser 727)	Cell Signaling Technologies	9134	Immunoblot	
	Danvers, MA, USA			
Rabbit anti-STAT3 mAb (D3Z2G)	Cell Signaling Technologies	12640	Immunoblot	
	Danvers, MA, USA			

**Secondary**				
Alexa-488 F(ab’)_2_ goat anti-rabbit IgG (H+L)	Life Technologies Inc	A11070	IF	
	Burlington ON, Canada			
Alexa-488 F(ab’)_2_ rabbit anti-rat IgG (H+L)	Life Technologies Inc	A21210	IF	
	Burlington ON, Canada			
Alexa-568 goat anti-rat IgG (H+L)	Life Technologies Inc	A11077	IF	
	Burlington ON, Canada			
Alexa-594 streptavidin	Life Technologies Inc	S32356	IF	
	Burlington ON, Canada			
HRP donkey anti-mouse IgG	Jackson Immunoresearch Laboratories Inc.	715-035-150	Immunoblot	
	West Grove PA, USA			
HRP donkey anti-rabbit IgG	Jackson Immunoresearch Laboratories Inc.	711-035-152	Immunoblot	
	West Grove PA, USA			

### 2.2 Mice

All procedures involving mice were approved by McMaster University׳s Animal Research Ethics Board in accordance with Canadian Council on Animal Care guidelines. Sources of mice were described in reference [1].

### 2.3 Bone marrow transplantation and evaluation of tunicamycin-induced apoptosis in vivo

10-week old male *wt* or *ldlr*^*-/-*^ mice were transplanted with *wt* or *pdzk1*^*-/-*^ bone marrow (BM) as described in the methods section of the accompanying paper [1]. For *ldlr* KO mice, four weeks after BM transplantation (BMT), atherosclerosis was induced by feeding the mice a high fat diet for 10 weeks as described in the accompanying paper [1]. For *wt* mice transplanted with either *wt* or *pdzk1*^-/-^ BM, BMT was carried out as described [1]. Eight weeks after BMT, mice were injected intraperitoneally with thioglycollate and, 72 h later, with tunicamycin (1 mg/kg body weight in 150 mM dextrose) as described in the methods section of the accompanying paper [1]. Mice were euthanized 24 h after tunicamycin injection, peritoneal cells were collected and analyzed by flow cytometry by staining for the myeloid marker CD11b, for apoptosis by staining for CC3 and propidium iodide as described in the methods section of the accompanying paper [1].

### 2.4 Blood cell analysis

Blood was collected by cardiac puncture into heparinized tubes. Red blood cell parameters (hematocrit, mean cell volume and distribution width) were determined using a Hemavet Multi-species Hematology System (Drew Scientific, Miami Lakes, FL, USA). For flow cytometry analysis of leukocytes, erythrocytes were lysed by incubating 0.2 mL of blood with 2.0 mL of 1× Flow Cytometry Mouse Lysis Buffer (R&D Systems, Minneapolis, MN, USA) for 10 min at room temperature. Afterwards, cells were pelleted (1200 rpm for 5 min in a microfuge at 4 °C), washed twice with FACS buffer (PBS containing 1% BSA) and labeled by incubation on ice for 1 hr with the following antibodies diluted 25-fold in FACS buffer: either FITC-labeled rat anti-mouse CD3, or both PerCP-Cy5.5 anti-hu/mo CD45R/B220 and APC anti-mouse CD11b. Flow cytometry was performed using a BD FACScalibur^TM^ flow cytometer (BD Biosciences, San Jose, CA, USA). Data was processed by FlowJo data analysis software (FlowJo, LLC., Ashland, OR, USA).

### 2.5 Monocyte Recruitment

Monocyte recruitment into atherosclerotic plaques was analyzed by labeling circulating monocytes with fluorescent beads, as previously described [7], [8]. *Ldlr* KO mice that had been transplanted with BM from *wt* or *pdzk1* KO donors and then fed the high fat, atherogenic diet for 10 weeks, as described in the accompanying paper [1], were injected i.v. with 250 μl PBS containing 1.5 × 10^11^ Fluoresbrite^®^ YG microspheres (0.5 μm, Polysciences, Inc., Warrington, PA, USA). 24 h after injection, mice were euthanized, and hearts were harvested and frozen in Shandon Cryomatrix (Thermo Fisher Scientific, Ottawa, ON, Canada). 10 μm transverse cryosections of aortic sinus were stained with oil red O. Fluorescence and brightfield images were captured using a Zeiss Axiovert 200 M inverted fluorescence microscope (Carl Zeiss Canada Ltd. Toronto, ON, Canada). The number of green fluorescent beads were quantified as previously described [8].

### 2.6 Immunofluorescence staining for KDEL and Ki67 in atherosclerotic plaques

To determine ER stress in macrophages in atherosclerotic plaques, ER chaperone proteins were detected with a mouse anti-KDEL mAb using Vector®M.O.M.™ immunodetection kit (Vector Laboratories, Inc., Burlingame, CA, USA) with an Alexa-594 streptavidin secondary reagent. Macrophages were stained with rat anti-mouse CD107b (Mac3) antibody followed by Alexa-488 labeled goat anti-rat antibody. Cell proliferation was determined by staining atherosclerotic plaques with rabbit monoclonal (SP6) Ki67 antibody, followed by Alexa-488 labeled goat anti-rabbit secondary antibody. Sections were also co-stained with DAPI to visualize nuclei. Fluorescent images were captured using a Zeiss Axiovert 200 M inverted fluorescence microscope (Carl Zeiss Canada Ltd. Toronto, ON, Canada).

### 2.7 Preparation, culture and treatment of peritoneal macrophages

Thioglycollate elicited peritoneal macrophages were prepared from mice as described [1]. Cells (1.5 × 10^5^ /well) were cultured in 8-well Nunc Lab-Tek II Chamber Slides (Thermo Scientific, Waltham, MA, USA) and treated with different agents as described [1]. Agents and concentrations used included: tunicamycin (10 μg/ml); oxLDL (100 μg protein/ml); HDL (50 50 μg protein/ml). Controls contained an equivalent amount of vehicle (0.1% DMSO for tunicamycin or saline for oxLDL). For UV irradiation, cells in chamber slides (with lids removed) were exposed to 50 mJ/cm^2^ of UV irradiation using a UVC-508 UV Crosslinker (Ultralum Inc, Clairmont CA, USA). Immediately following UV irradiation, cell culture media was replaced with fresh media containing or lacking HDL at the concentrations indicated and cells were cultured for 24 h prior to apoptosis analysis by TUNEL staining as described [1].

### 2.8 Immunoblotting

Cells were treated as described in ref [1]. Briefly, for phosphoprotein analyses, cells were serum starved for 2 h prior to HDL addition. Cells were lysed on ice with RIPA buffer (50 mM Tris–HCl pH7.4; 150 mM NaCl; 1% Triton X-100; 1% sodium deoxycholate; 0.1% SDS; 1 mM EDTA) in the presence of protease inhibitors (1 μg/ml pepstatin A; 1 μg/ml leupeptin; 10 μg/ml aprotinin; 50 μM APMSF) and PhosSTOP phosphatase inhibitor cocktail (Roche, Mannhein, Germany). Protein concentrations in clarified supernatants were determined (BCA assay, Pierce Biotechnology, Rockford, IL, USA) and 20–50 μg proteins were subjected to SDS-polyacrylamide (15%) gel electorphoresis and immunoblotting on PVDF. Membranes were blocked (1 hr, room temp.) with 5% skim milk in TBS+0.1% Tween-20 (blocking solution), incubated with primary antibodies (4 °C overnight) and secondary antibodies (1 hr at room temp) diluted as indicated, with washes in TBS+0.1% Tween-20 in between. Primary antibodies were rabbit anti-phospho(Y705)-STAT3, rabbit anti-STAT3, rabbit anti-phospho-MLKL (each diluted 1:1000 in TBS-T with 3% BSA) and mouse anti-mouse KDEL (1:1000 dilution in blocking solution). Secondary antibodies were HRP-donkey anti-mouse IgG and HRP-donkey anti-rabbit IgG (each 1:10000 in blocking solution). Blots were stripped and re-probed using HRP-anti-GAPDH or HRP-anti-β-actin antibodies (1:5000 in blocking solution, 1 hr at room temp.) as loading controls. HRP was detected using Amersham Enhanced Chemiluminescence reagents (GE Healthcare Life Sciences, Baie d’Urfe, QC, Canada) and a Gel Doc instrument (Bio-Rad Laboratories, Hercules, CA, USA).

### 2.9 RT-PCR

Cells were treated with 10 ng/ml LPS for 6 h. Total RNA purification, quantification and cDNA synthesis was as described in ref [1]. Real-time quantitative PCR was performed using Platinum Sybr Green dye (Invitrogen Life Technologies Inc., Burlington, ON, Canada) in an Applied Biosystems 7300 Real Time PCR system (Applied Biosystems, Foster City, CA, USA) with default settings. All primers (Table 3) were synthesized by Life Technologies (Burlington, ON, Canada).

**Table 3:** Primers used for RT-PCR.

**Genes**	**Primer sequences**	**References**
CHOP	Forward: 5′-CCTAGCTTGGCTGACAGAGG-3′	[9]
	Reverse: 5′-CTGCTCCTTCTCCTTCATGC-3′	
		
IL-1β	Forward: 5′- AGGCAGGCAGTATCACTCATTGT-3′	[10]
	Reverse: 5′- GGAAGGTCCACGGGAAAGA-3′	
		
IL-6	Forward: 5′-TAGTCCTTCCTACCCCAATTTCC-3′	[11]
	Reverse: 5′- TTGGTCCTTAGCCACTCCTCC-3′	
		
MCP-1	Forward: 5′- TTCCTCCACCACCATGCAG-3′	[12]
	Reverse: 5′- CCAGCCGGCAACTGTGA-3′	
		
GAPDH	Forward: 5′-ACCACAGTCCATGCCATCAC-3′	[13]
	Reverse: 5′-TCCACCACCCTGTTGCTGTA-3′	

### 2.10 Statistical analysis

Statistical analysis was performed using Prism software (GraphPad Software, San Diego, CA, USA). The Mann-Whitney rank sum test was used for analysis of data from two groups and one-way or two-way ANOVA with Tukey׳s multiple comparisons test was used for more than two groups. Data are presented as mean ± SEM and were considered statistically significant if *p* < 0.05.
