# Supplementary material for: Data on leukocyte PDZK1 deficiency affecting macrophage apoptosis but not monocyte recruitment, cell proliferation, macrophage abundance or ER stress in atherosclerotic plaques of LDLR deficient mice
Source: Data Brief. 2018 May 26;19:1148–61. doi: 10.1016/j.dib.2018.05.128 (PMC6141767; doi:10.1016/j.dib.2018.05.128)
Supplement: Supplementary file 1 — Supplementary material [file mmc1.pdf]

On behalf of all authors, Bernardo L. Trigatti wishes to confirm that there are no known conflicts of interest associated with this publication and there has been no significant financial support for this work that could have influenced its outcome.
